# Identification and Functional Characterization of Plant Toxins

**DOI:** 10.3390/toxins13030228

**Published:** 2021-03-22

**Authors:** Zbigniew Adamski, Sabino Aurelio Bufo, Luigi Milella, Laura Scrano

**Affiliations:** 1 Laboratory of Electron and Confocal Microscopy, Faculty of Biology, Adam Mickiewicz University in Poznań, ul. Uniwersytetu Poznańskiego 6, 61-614 Poznań, Poland; 2 Department of Animal Physiology and Development, Institute of Experimental Biology, Adam Mickiewicz University in Poznań, ul. Uniwersytetu Poznańskiego 6, 61-614 Poznań, Poland; 3 Department of Sciences, University of Basilicata, Via dell’Ateneo Lucano 10, 85100 Potenza, Italy; 4 Department of Geography, Environmental Management & Energy Studies, University of Johannesburg, Auckland Park Kingsway Campus, Johannesburg 2092, South Africa; 5 Department of European and Mediterranean Cultures, University of Basilicata, Via Lanera 20, 75100 Matera, Italy

The evolutionary arms race between plants and herbivores has led, over millions of years, to the production of many substances that prevent plants from being over-eaten by plant-feeding animals. These substances have a toxic or repellent effect, reducing plant pathogens’ viability and abundance, limiting plant feeding and, consequently, decreasing plant biomass loss. Many plant constituents can be toxic to herbivores and plant pathogens, and other organisms, such as viruses, bacteria, fungi, other plants, and vertebrates, including humans. For centuries, such substances have been part of humankind’s history, often being the cause of accidental poisonings known from history, deliberate poisonings, or executed sentences. However, such substances may also have a bright side: they can combat pathogens, treat many diseases, including cancer, and fight microorganisms harmful to humans. In plant-cropping conditions, such compounds can undoubtedly control pests of crops and food products [1]. The current Special Issue collects publications on the identification and functional characterization of toxic substances produced by plants. The readers can distinguish several articles trends: (i) identification (using various chemical and biochemical methods), (ii) biological activities, (iii) and the possible application of plant-produced substances. Often, they are amalgamated in the same manuscripts. We believe that such a strategy, combining the description of plant substances and their possible application, is interesting and sets future research directions. Feldberg and co-workers [2] described the applicability of an assay for the identification of ricin—obtained from castor bean plant (*Ricinus communis* L.)—in body fluids using mass spectrometry (MS). The described sensitive method can detect ricin down to 5 ng/mL in serum samples. The method was successful in clinical scenarios, too. Quinolizidine alkaloids, substances produced by leguminous plants, were the next paper’s topic [3]. The authors precisely described their investigations to optimize the chromatographic system for the analysis of cytisine and *N*-methylcytisine in plant extracts obtained from various Fabaceae family plants. They also described the cytotoxic activity of extracts and identified the *Genista germanica* L. leaves extract and *Laburnum watereri* (Wettst.) Dippel seed extract as the most active against selected cell lines. Not only dicotyledonous plants produce active substances. They can be found in monocotyledonous plants, too. The perennial ryegrass (*Lolium perenne* L.), in response to infection by a fungus *Epichloë festucae*, produces epoxyjanthitrems and epoxyjanthitriol. The next manuscript in the volume reports a full structural elucidation yielding NMR (nuclear magnetic resonance) assignments for these compounds [4]. In the paper are also reported the substances toxic against invertebrates and vertebrates. The toxicity of plant-derived substances against pests is a topic that attracts numerous groups of researchers. Ntalli et al. [5] described *Stevia rebaudiana* (Bertoni) Bertoni water extracts’ chemical composition against important agricultural pests as nematodes—*Meloidogyne incognita* and *Meloidogyne javanica*—and proved that the extracts and leaves’ powder might successfully control them. To the authors’ knowledge, they reported, for the first time, some metabolites in Stevia species. Moreover, the applied powder was not phytotoxic. Such a lack of activity against plants is crucial for agriculture. Bajwa et al. [6] discussed the importance of secondary metabolites of parthenium weed (*Parthenium hysterophorus* L.), an invasive plant, in their invasion success. They also estimated it as limited. The authors also described and compared phytotoxic, cytotoxic, and phyto-cytotoxic activity of leaf- shoot- and root-extracts. Hence, both the abovementioned manuscripts fit into the research pattern showing the importance of research that compares toxic activity for various species, including pests and beneficial ones. Interestingly, even if the effects caused by the toxic plant-derived substances are limited to sublethal ones, they may significantly limit the emission of synthetic insecticides in the environment. Spochacz and coworkers [7] proved the proper strategy combining the emission of plant-derived substances and synthetic insecticides. The first one, emitted before but not together with the synthetic insecticide, significantly increases the latter’s toxicity. In consequence, its spread to the environment can be significantly lower. The amount of research on plant-derived substances increases rapidly. Many papers showing the composition of plant extracts, essential oils, powders, single compounds, their biological activity, application in agriculture and medicine are published each month. The review papers presenting the state-of-the-art in the field summarize the recent steps in this field. Therefore, we are proud to present two review papers offering the current knowledge of plant-derived substance effects in this volume. The first one describes *Cannabis sativa* L. as a source of phytocannabinoids, distribution of endocannabinoids receptors through the human body, the biological activity of these substances, and their potential therapeutic effects [8]. The authors emphasize that *C. sativa* has potential therapeutic properties, but there are also tendencies of abuse. Therefore, the activity of cannabinoids needs to be still intensively studied, and application must be carefully monitored. Additionally, berberine isoquinoline alkaloids show high therapeutic potential. Its anticancer, proapoptotic, and anti-inflammatory activity has been described [9]. The compound is regarded as one of the most promising substances in breast and colon cancers. The presented papers show the vast gamut of plant-derived substances and the range of their possible usage in pure science, medical studies, plant protection, and evolutionary relationships. We believe that the presented papers increase our knowledge within the field. They will contribute to the intensive development of science concerning plant-derived substances’ activities and their possible usage in the future.
